# Dynamic Recrystallization Behaviours in Metals and Alloys

**DOI:** 10.3390/ma16030976

**Published:** 2023-01-20

**Authors:** Frank Montheillet

**Affiliations:** CNRS, UMR 5307 Laboratoire Georges Friedel, Centre SMS, Mines Saint-Etienne, 158 cours Fauriel, CEDEX 2, 42023 Saint-Etienne, France; montheil@emse.fr

The existence of dynamic recrystallization (DRX), i.e., recrystallization occurring during straining, has long been questioned [1] despite the publication of strong mechanical and microstructural evidence [2]. Some authors later showed that it was not merely a “laboratory curiosity” but in fact a real “industrial tool” [3]. Currently, DRX has been defini tively recognized as the most important physical mechanism associated with the hot working of metals and alloys, an understanding of which is key to the optimization of microstructural and mechanical properties.

Although DRX was first imagined to take place exclusively in low to medium stacking fault energy (SFE) materials, it was later observed that high SFE metals, such as ferritic steels or aluminium alloys, also exhibit recrystallization-like microstructure transformations during hot working. In the first case, DRX occurs by nucleation and the growth of new grains, which has been termed discontinuous DRX (DDRX); in the second case, DRX results from the progressive “fragmentation” of the initial grains and is often referred to as continuous DRX (CDRX) [4]. The aim of this Special Issue is to present recent novel research on this wide topic. The behaviour of a variety of alloys submitted to new hot-working processes (Figure 1) and/or with new compositions is addressed, which highlights the importance of DRX in the whole field of the thermomechanical processing of metals (Figure 2).

Nagira et al.’s paper [5] investigates the DRX of both commercial-grade and high-purity aluminium occurring during friction stir welding; possible transitions between DDRX and CDRX are revealed, which are associated with distinct texture components. Dolzhenko et al.’s article [8] deals with DRX in an austenitic stainless steel containing about 10 vol% ferrite together with a small fraction of nanometric Z-phase (CrNbN) particles submitted to compression tests. Power-law functions are used to relate the various mechanical and microstructural parameters to each other.

Two contributions are devoted to near-β titanium alloys deformed in uniaxial compression in both the α and α + β domains: Zhou et al. [10] investigate a low-cost iron-containing alloy, while Buzolin et al. [11] develop mesoscale models to predict the flow stress and microstructure evolutions of the Ti-5553 and Ti-17 grades. Microstructure and texture evolutions of a new Zr-Ti-Al-V alloy are investigated by Lei et al. [9], who point out the co-existence of DDRX and CDRX.

Three papers deal with the hot working of magnesium alloys, which are much less frequently mentioned in the literature. Two similar Mg-Gd-Y alloys are submitted to large strains by Wu et al. [6] and Liu et al. [7] using reciprocating upsetting-extrusion and multi-directional forging deformation processes, respectively. In a similar way, Ullmann et al. [12] investigate a twin-roll-cast Mg-Y-Zn alloy by plane-strain compression. The articles converge on the conclusion that complex CDRX and/or DDRX mechanisms lead to grain refinement and texture weakening, thus improving formability.

Finally, a theoretical paper by Montheillet [13] points out the importance of the softening induced by grain boundary migration (BMIS) during DDRX, in particular for the estimation of grain boundary mobility from experimental data.

## Figures and Tables

**Figure 1:**
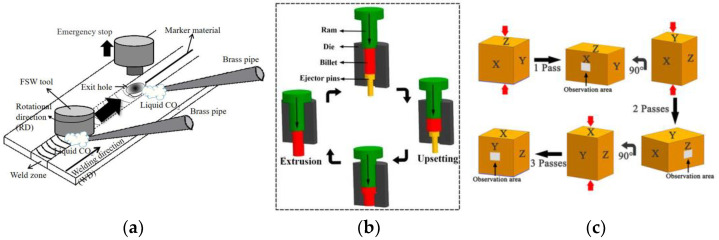
Schematic representations of some processes used to prescribe large-strain hot deformations: (**a**) friction stir welding [5]; (**b**) reciprocating upsetting-extrusion [6]; (**c**) multidirectional forging [7].

**Figure 2:**
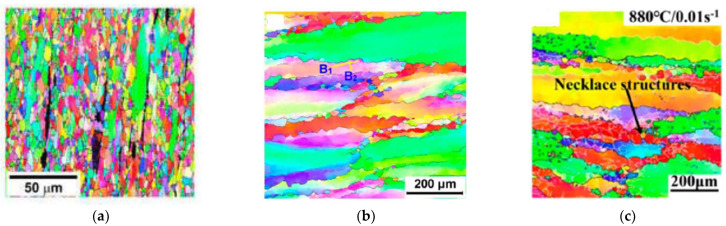
Examples of microstructures exhibiting DRX in various alloys: (**a**) DDRX in a highly alloyed austenitic stainless steel (1000 °C, 10 ^−4^ s ^−1^, ε = 1) [8]; (**b**) onset of CDRX/DDRX in a Zr-Ti-Al-V alloy (800 °C, 10 ^−2^ s ^−1^, ε = 0.7) [9]; (**c**) onset of CDRX in Ti-35421 (880 °C, 10 ^−2^ s ^−1^, ε = 0.9) [10].
